# Promoting Brain Health and Resilience in Family Caregivers of Adults with Intellectual and/or Developmental Disabilities

**DOI:** 10.1002/alz70860_104552

**Published:** 2025-12-23

**Authors:** Yona Lunsky, Mary Chiu, Anupam Thakur, Prachi Patel, Nicole Bobbette, Tiziana Volpe, Robert Balogh, Lee Steel, Amy Baskin, Anna Lobo, Johanna Lake, Brain Health IDD team

**Affiliations:** ^1^ Centre for Addiction and Mental Health, Toronto, ON, Canada; ^2^ Ontario Shores Centre for Mental Health Sciences, Whitby, ON, Canada; ^3^ CAMH, Toronto, ON, Canada; ^4^ Queen's University, Kingston, ON, Canada; ^5^ Ontario Tech University, Oshawa, ON, Canada

## Abstract

Adults with intellectual and/or developmental disabilities (IDD) face increased risks of age‐related health issues, including dementia, yet current brain health initiatives often overlook the needs of this unique population. To address this gap, our team developed the *Brain Health‐IDD* program ‐ a co‐designed virtual education initiative aimed at promoting brain health among aging adults with IDD (aged 40+), and family caregivers. The program utilizes an integrated Knowledge Mobilization (KMb) approach, engaging individuals with lived experience, community members, and professionals throughout the co‐design process to ensure relevance and inclusivity.

Family caregivers of individuals with IDD are at a heightened risk of developing dementia themselves, due to the chronic stress associated with the caregiver role. Although some supports focus on assisting aging parents and siblings supporting these individuals, minimal attention has focused on the long term impacts of caregiving on their brain health. The *Brain Health‐IDD* Family course aims to mitigate this risk by providing caregivers with critical information on brain health, stress management, and strategies for improving their own cognitive and physical well‐being. By improving caregiver self‐efficacy, the program seeks to reduce stress, promote resilience, and ultimately improve the long‐term health outcomes for caregivers, in addition to the individuals they support. Because the program is virtual, it is available to aging family caregivers from across Canada, and not reliant on local expertise.

The *Brain Health‐IDD* Family course consists of 6 weekly 90‐minute virtual sessions, co‐led by interdisciplinary teams of caregivers, brain health experts and clinician scientists. The primary objectives of the program are to encourage changes in health behaviors, physical and mental well‐being, and the initiation of cognitive screening among participants. To evaluate impact of our Program, we use a mixed‐methods, pre‐post study to collect caregivers’ outcomes at baseline, 7 weeks (post‐intervention), and 18 weeks. Data from satisfaction surveys, qualitative interviews, and open‐text surveys from the first cohort have informed the iterative refinement of course content and delivery. The third cohort will participate in the program between February and March of 2025, with analysis completed on over 100 aging family caregivers by summer of 2025.

